# Maternal Plasma Proteomic Signatures Distinguish Preeclampsia from Healthy Pregnancy but Not Early-Onset from Late-Onset Subtypes: A Leakage-Free Machine-Learning and Explainability Study

**DOI:** 10.3390/ijms27209275

**Published:** 2026-10-19

**Authors:** Seyma Yasar, Rauf Melekoglu, Nese Basak Turkmen, Aishana Safarova, Cemil Colak, Sarah A. Alzakari, Amal K. Alkhalifa, Fatma Hilal Yagin

**Affiliations:** 1 Department of Biostatistics and Medical Informatics, Faculty of Medicine, Inonu University, Malatya 44280, Türkiye; 2 Department of Obstetrics and Gynecology, Faculty of Medicine, Inonu University, Malatya 44280, Türkiye; rauf.melekoglu@inonu.edu.tr (R.M.);; 3 Department of Pharmaceutical Toxicology, Faculty of Pharmacy, Inonu University, Malatya 44280, Türkiye; 4 Department of Computer Sciences, College of Computer and Information Sciences, Princess Nourah Bint Abdulrahman University, P.O. Box 84428, Riyadh 11671, Saudi Arabia; 5 Department of Biostatistics and Medical Informatics, Faculty of Medicine, Balikesir University, Balikesir 10145, Türkiye

**Keywords:** preeclampsia, early-onset, late-onset, plasma proteomics, LC–MS/MS, machine learning, nested cross-validation, SHAP, LIME, data leakage, biomarkers

## Abstract

Early-onset (EOPE) and late-onset (LOPE) preeclampsia are clinically distinct, but their molecular differences are incompletely defined. Using a strictly leakage-free pipeline, we re-examined maternal plasma proteomes of EOPE, LOPE, and healthy pregnancy (HP) and asked whether the subtypes are separable at the proteome level. Label-free LC–MS/MS proteomes comprising 370 proteins from 46 women (HP, *n* = 18; EOPE, *n* = 17; LOPE, *n* = 11) were analysed using limma and fully nested cross-validation, in which feature selection, class balancing, and tuning were confined to the outer training folds; model explanations used SHAP and feature-selection stability. Observed selection frequencies far exceeded a random-selection null (for example, complement factor D was selected in all 15 outer folds, Wilson 95% confidence interval 0.80 to 1.00, against an expected frequency of 0.07), indicating that the stability was not a chance artefact. Discrimination is summarised as the mean ± standard deviation across the 5-fold × 3-repeat (15) outer test folds; because the single best-performing classifier was chosen on outer-fold performance, the reported area under the curve is modestly optimistic (on average by 0.04, and by up to 0.10 relative to the median of the three classifiers), even though the leakage-free procedure was applied identically to each classifier. Disease-versus-healthy contrasts were strong and concordant, with 10 and eight differentially abundant proteins identified for EOPE and LOPE, respectively, and AUROCs ranging from 0.88 to 0.90. EOPE showed a complement/acute-phase/coagulation signature, whereas LOPE showed an apolipoprotein/lipid signature, with limma, stability analysis, and SHAP yielding convergent findings. By contrast, the EOPE-versus-LOPE comparison identified no differentially abundant proteins and showed no reliable multivariate separation. A post hoc sensitivity analysis (two-sample *t*-test, two-sided, α = 0.05) indicated that the discovery cohort (early-onset *n* = 17, late-onset *n* = 11) had 80% power only for very large per-protein standardised differences (Cohen’s d ≥ 1.13, or d ≥ 2.10 after Bonferroni correction across the 370 proteins), and only 24% and 51% power to detect medium (d = 0.5) and large (d = 0.8) effects; the absence of a detectable early-versus-late difference is therefore consistent with limited power for small-to-moderate effects rather than proven biological equivalence. In an independent ELISA cohort (*n* = 87), EOPE differed from HP for seven of nine analytes, with AUCs of up to 0.82, led by sFlt-1 (fold-changes paralleled these areas under the curve: the largest early-onset median disease-to-control ratios were fibronectin 2.9, soluble fms-like tyrosine kinase-1 2.8 and α1-antitrypsin 2.5, whereas placental growth factor decreased to 0.6 and apolipoprotein E was unchanged at 1.0); however, the subtypes again did not separate. On adjustment for maternal age, body mass index and smoking, the leading early-onset markers remained significant (soluble fms-like tyrosine kinase-1, serum amyloid A and fibronectin robust; complement factor D and alpha-1-antitrypsin borderline), whereas maternal chronic hypertension, diabetes and thyroid disease were not recorded and could not be modelled. Benchmarked against soluble fms-like tyrosine kinase-1 (DeLong test), no novel candidate exceeded its discrimination and several were significantly lower; only glycoprotein hormones alpha chain added cross-validated incremental value in early-onset disease (cross-validated area under the curve 0.71 to 0.76), and none improved on soluble fms-like tyrosine kinase-1 in late-onset disease; apolipoprotein E did not validate, and placental growth factor decreased as expected. Plasma proteomics distinguishes preeclampsia from healthy pregnancy and recovers coherent subtype-leaning signatures but provides little evidence of a sharp EOPE–LOPE boundary; the prioritised panel warrants validation in larger cohorts.

## 1. Introduction

Preeclampsia is among the most consequential disorders of pregnancy: it accounts for a substantial share of maternal and perinatal death, its only definitive treatment is delivery, and women who experience it carry an elevated cardiovascular risk for decades afterwards [1,2]. It complicates roughly 2–8% of pregnancies, yet no molecular tool reliably identifies the disease early or explains why affected women follow such divergent clinical courses [1].

By the gestational age at which it declares itself, the disorder is divided into early-onset (before 34 weeks) and late-onset (from 34 weeks) forms, a distinction that carries prognostic rather than merely descriptive weight. Early-onset disease is more often accompanied by deficient spiral-artery remodelling, placental insufficiency and foetal growth restriction, whereas late-onset disease tends to arise on a background of maternal metabolic and vascular susceptibility with a comparatively preserved placenta [1,3]. This contrast is usually read through the two-stage placental model, in which impaired placentation precedes a systemic maternal syndrome [3,4]. Whether the two onset forms are mechanistically separate or occupy two ends of a continuum remains unsettled, and the answer bears directly on how prediction and prevention should be targeted.

The angiogenic imbalance between soluble fms-like tyrosine kinase-1 (sFlt-1) and placental growth factor (PlGF) is the best-established molecular hallmark of the syndrome. Circulating sFlt-1 rises and PlGF falls before symptoms appear, and the sFlt-1/PlGF ratio has entered clinical practice, chiefly to rule out disease over the short term [5]. Its performance is strongest for early-onset preeclampsia, and a single ratio cannot capture the broader, subtype-specific protein changes that accompany the disease [1,5].

Untargeted plasma proteomics offers a route to that wider picture, and several groups have reported candidate signatures separating preeclampsia from normotensive pregnancy [6,7,8]. Agreement between studies has been modest, partly because cohorts are small and differ in sampling time and clinical definition. Small proteomic datasets also expose machine-learning analyses to a well-characterised hazard: information leakage, in which feature selection, class balancing or hyperparameter tuning are permitted to see the test data and inflate apparent performance. This problem has been documented across hundreds of studies in seventeen disciplines [9], and it is most dangerous when the contrast of interest—one preeclampsia subtype against the other—is subtle.

We revisited an existing maternal plasma proteomic dataset of early-onset, late-onset and normotensive pregnancies [8] under a deliberately leakage-free design. Differential abundance was estimated with robust moderated statistics [10], classification used fully nested cross-validation, and the resulting models were interpreted with two complementary explanation methods so that reproducible signal could be distinguished from an artefact. We set out to define disease-versus-healthy signatures and their subtype leanings, to test with both univariate and multivariate evidence whether early- and late-onset preeclampsia are separable in the circulating proteome, and to examine a prioritised, convergence-based protein panel in a separate cohort by ELISA.

## 2. Results

### 2.1. Cohort and Analysis Overview

The cohort and the three pairwise comparisons are summarised in Table 1. After preprocessing, approximately 250 proteins per comparison entered differential testing.

### 2.2. Disease-Versus-Healthy Proteomic Signatures

**Figure 1:**
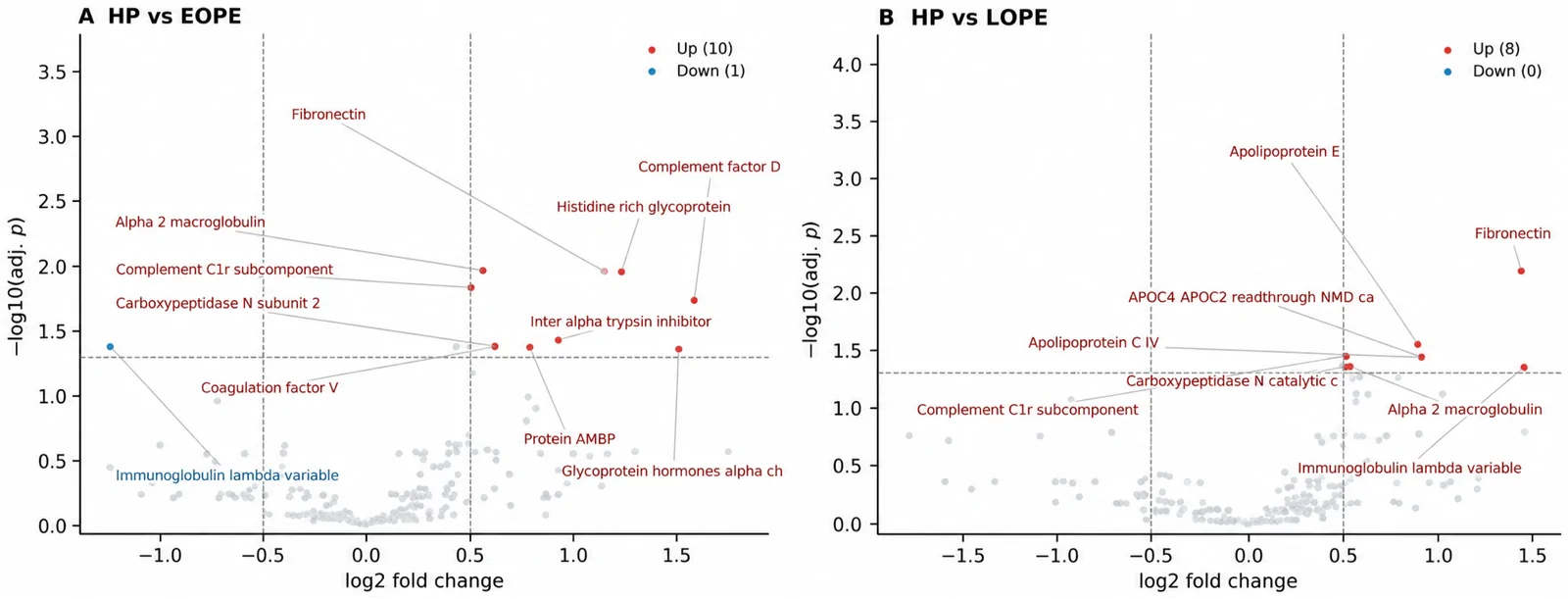
Volcano plots of differential abundance for (**A**) HP vs. EOPE and (**B**) HP vs. LOPE (limma robust empirical Bayes with independent filtering; dashed lines mark adj.*p* = 0.05 and |log2FC| = 0.5). Discovery cohort sizes: healthy pregnancy *n* = 18, early-onset *n* = 17, late-onset *n* = 11. Up-regulated proteins (adj.*p* < 0.05, |log2FC| > 0.5) are highlighted. The EOPE vs. LOPE comparison revealed no differentially abundant proteins (minimum adj.*p* ≈ 1.0). Grey dots represent proteins that did not reach statistical significance, whereas red and blue dots indicate significantly upregulated and downregulated proteins, respectively.

Both disease-versus-healthy contrasts produced strong, biologically coherent signatures, all up-regulated relative to healthy pregnancy (Table 2; Figure 1). HP vs. EOPE yielded 10 DEPs dominated by complement, acute-phase and coagulation proteins—including complement factor D, histidine-rich glycoprotein, α2-macroglobulin, complement C1r, inter-α-trypsin inhibitor heavy chain and coagulation factor V—consistent with the placental/inflammatory phenotype of early-onset disease. HP vs. LOPE yielded 8 DEPs led by fibronectin and an apolipoprotein/lipid cluster (apolipoprotein E, apolipoprotein C-IV, APOC4-APOC2), consistent with the maternal/metabolic phenotype of late-onset disease. Fibronectin and complement C1r/complement factor D recurred across both comparisons, indicating a shared endothelial–complement core.

Over-representation analysis of the differentially abundant proteins mirrored these signatures (Supplementary Table S2). For early-onset preeclampsia versus healthy pregnancy, complement and coagulation processes were enriched—KEGG complement and coagulation cascades, Reactome hemostasis and complement cascade, and the Hallmark coagulation and complement sets (all FDR < 0.05)—consistent with an acute-phase, coagulation-active profile. For late-onset preeclampsia, lipoprotein and cholesterol terms dominated, including Reactome plasma-lipoprotein assembly and remodelling, chylomicron assembly and liver-X-receptor-mediated cholesterol signalling, together with KEGG cholesterol metabolism and complement and coagulation cascades (all FDR < 0.05). Because these small differentially abundant sets (eight to ten proteins) were tested against a genome background, the enrichment is interpreted as descriptive support for the disease-versus-healthy biology rather than as an independent quantitative result.

### 2.3. Nested Cross-Validation Classification

Leakage-free nested-CV classification (Table 3) separated each disease from healthy pregnancy with AUROC 0.88–0.90, with CatBoost and XGBoost generally being the best. Boruta produced markedly more parsimonious and stable feature sets (≈28 proteins) than RFECV (≈54–68 proteins, large variance), with comparable performance, and is therefore used as the primary feature-selection method throughout. The EOPE-versus-LOPE comparison is treated separately in Section 2.5 and Supplementary Table S1.

### 2.4. Convergence of Differential Analysis, Selection Stability and SHAP

**Figure 2:**
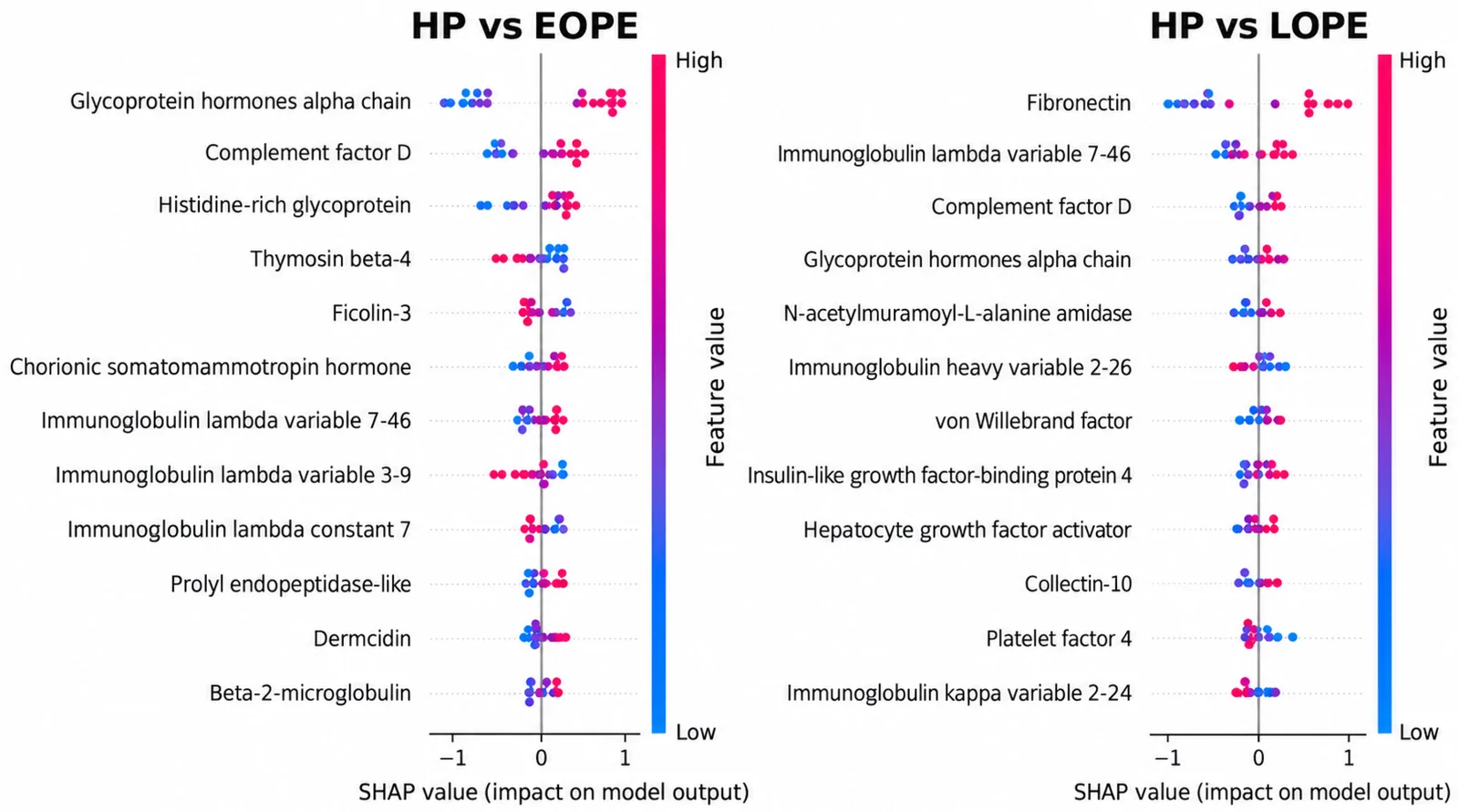
SHAP summary (beeswarm) plots of the best classifier (CatBoost, Boruta feature set) for (**left**) HP vs. EOPE and (**right**) HP vs. LOPE. SHAP, SHapley Additive exPlanations; the plotted features are the Boruta-selected proteins from leakage-free nested cross-validation. Each dot is a participant; horizontal position is the SHAP value (impact on the model output); and colour encodes the protein abundance (blue low, red high). Features are ordered by mean |SHAP|. For EOPE, high glycoprotein hormones alpha chain, complement factor D and histidine-rich glycoprotein push predictions toward disease; for LOPE, fibronectin dominates. EOPE vs. LOPE is omitted as its model carried no coherent signal.

**Figure 3:**
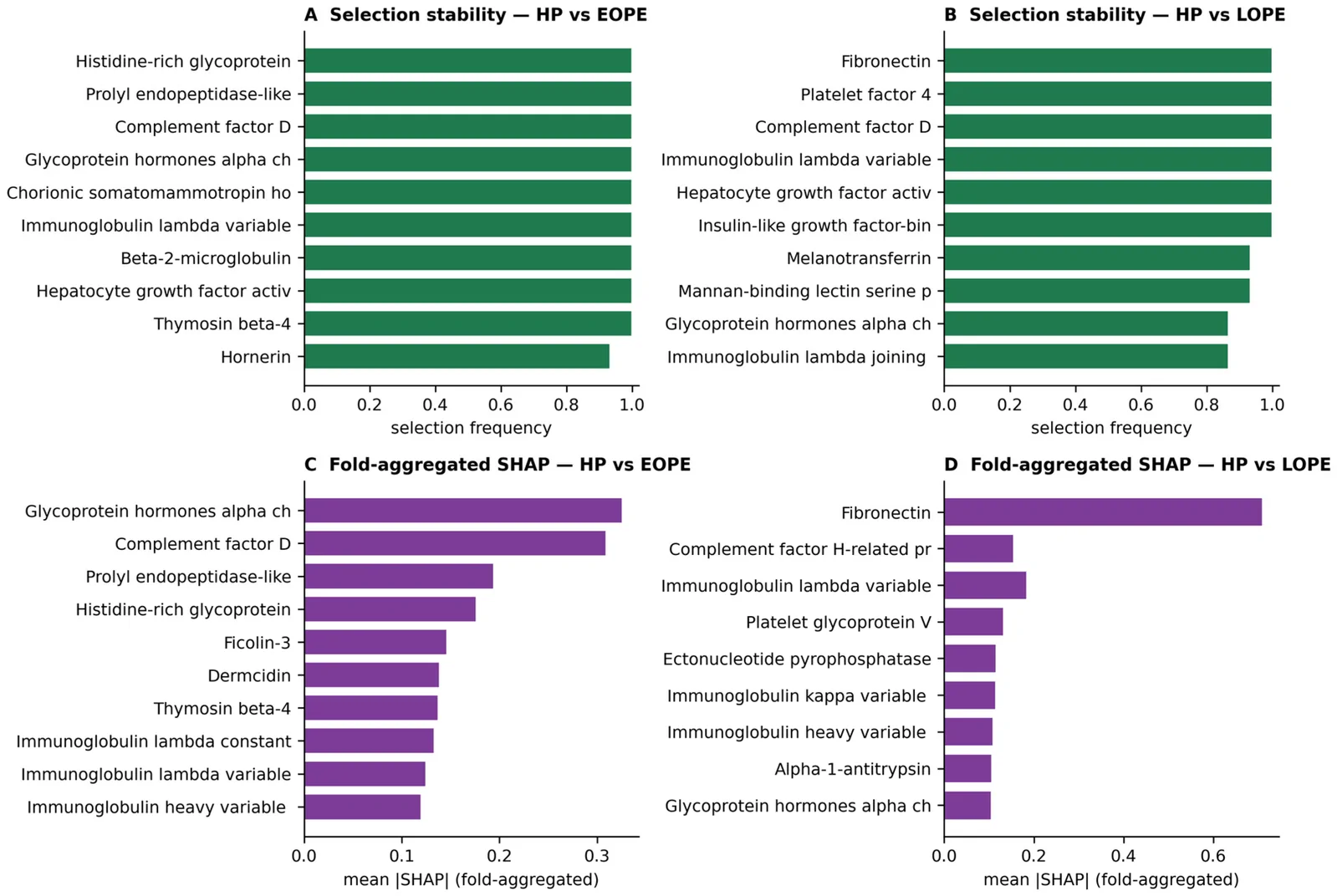
Convergence of feature-selection stability and leakage-free explanation. (**A**,**B**) Selection frequency across outer folds (nested cross-validation, Boruta). (**C**,**D**) Fold-aggregated SHAP importance (Method 2), computed on held-out outer-test partitions, for HP vs. EOPE and HP vs. LOPE. The same core proteins recur across stability and SHAP, supporting their robustness.

**Figure 4:**
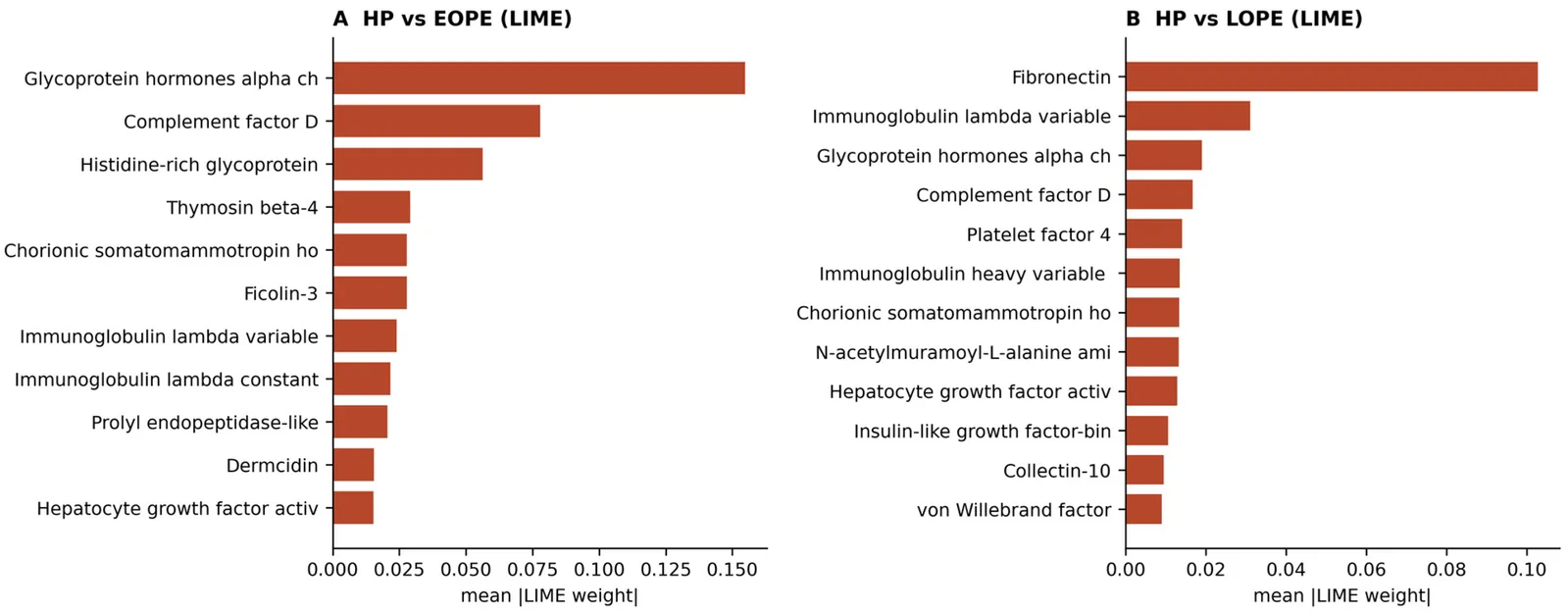
LIME aggregate importance (mean absolute local weight across participants) for the best classifier of (**A**) HP vs. EOPE and (**B**) HP vs. LOPE. The LIME rankings reproduce the SHAP findings—glycoprotein hormones alpha chain, complement factor D and histidine-rich glycoprotein for EOPE, and fibronectin for LOPE—demonstrating that the explanations are stable across two complementary post hoc explanation methods.

**Figure 5:**
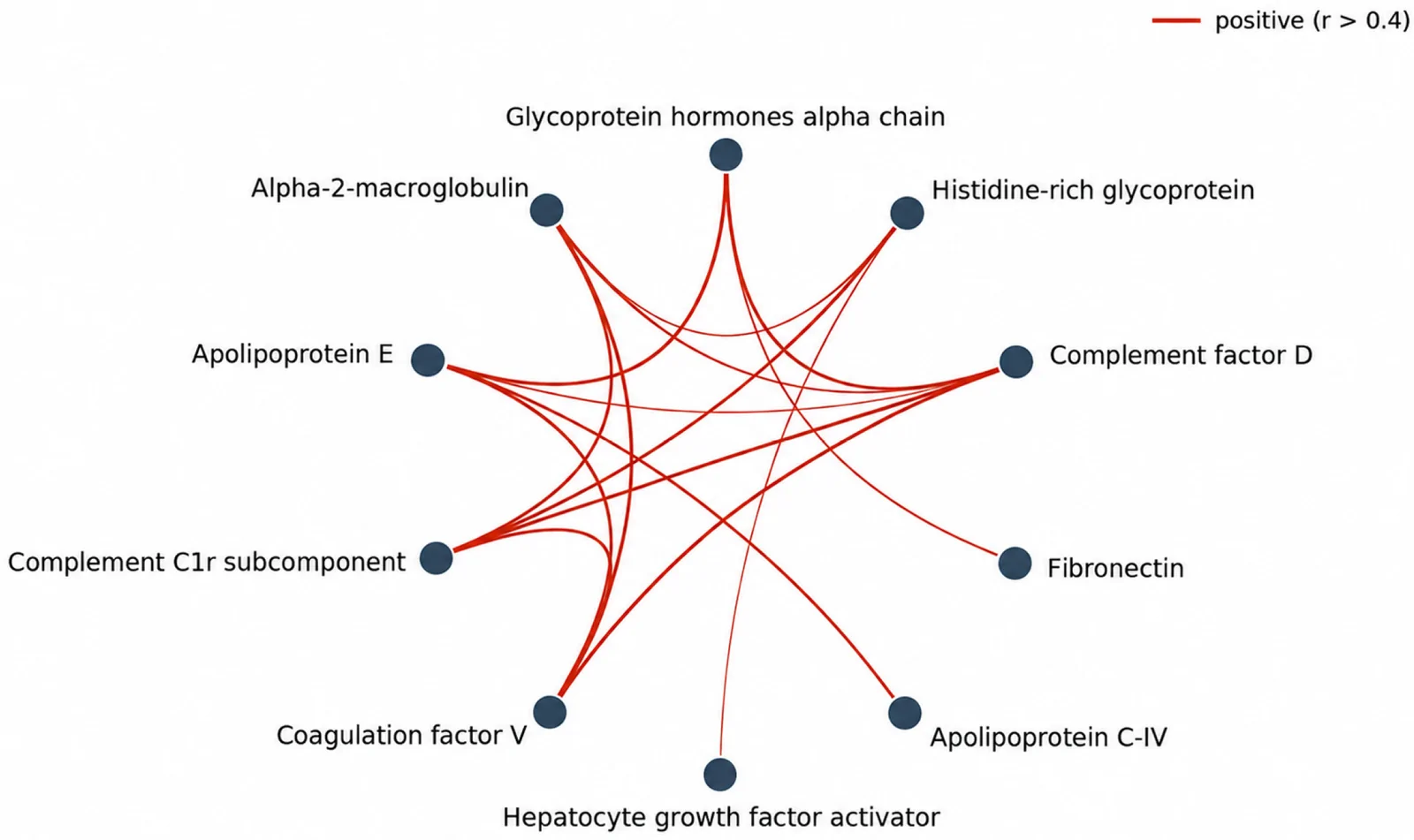
Co-abundance structure of the top convergent proteins across the whole cohort. Nodes are proteins arranged on a circle; edges connect protein pairs with |Pearson r| > 0.4 (red, positive), with width proportional to correlation strength. A tightly co-abundant complement/acute-phase/coagulation module (α2-macroglobulin, complement C1r, complement factor D, coagulation factor V, apolipoprotein E) is evident, consistent with coordinated abundance patterns across these proteins; because whole-cohort correlations can be inflated by shared between-group differences, this is described rather than interpreted as pathway activation.

The three evidence streams—limma DEPs, nested-CV selection stability and SHAP importance—converged on the same core markers. For HP vs. EOPE, complement factor D and histidine-rich glycoprotein were simultaneously DEPs, stably selected and high-SHAP; glycoprotein hormones alpha chain (CGA) was the top SHAP feature by both methods and stably selected, although it was not a top univariate DEP—an example of a multivariate signal recovered by the model that the marginal test missed, biologically consistent with the placental phenotype of EOPE. For HP vs. LOPE, fibronectin was the leading DEP, the most stably selected feature and the dominant SHAP feature (Figure 2), giving four-way convergence (Figure 3); complement factor D again recurred. LIME, applied as a complementary post hoc explanation method, reproduced these rankings (Figure 4): the same top three proteins for EOPE and the same fibronectin dominance for LOPE, indicating that the explanations are method-robust rather than artefacts of a single algorithm. Finally, the leading convergent proteins formed a tightly co-abundant complement/acute-phase/coagulation module across the cohort (Figure 5), consistent with coordinated abundance patterns among these proteins. This concordance across methods provides internal support for the robustness of these markers despite the small discovery sample.

### 2.5. No Protein Distinguished EOPE from LOPE

No protein was differentially abundant between EOPE and LOPE at either the confirmatory or the discovery threshold (minimum adjusted *p* ≈ 1.0). Several factors explain this null. First, the subtype contrast is the smallest and most imbalanced (EOPE = 17, LOPE = 11), giving limited statistical power. Second, robust empirical Bayes moderation appropriately down-weighted hyper-variable immunoglobulin features, which are the only proteins that show large nominal between-subtype differences but reflect individual rather than disease-related variation. Third, and most informatively, the absence of a signal was not confined to the univariate analysis: leakage-free multivariate machine learning, which can capture multivariate effects that marginal tests miss (as it did for glycoprotein hormones alpha chain in EOPE), likewise failed to separate the subtypes reliably, and its explanations were immunoglobulin-dominated and method-inconsistent. The convergence of a univariate null and a multivariate null indicates that EOPE and LOPE are not sharply separable in the circulating proteome of this cohort, consistent with substantial overlap rather than discrete proteomic states. The detailed EOPE-versus-LOPE results (classification metrics, stability and explanations) are provided in Supplementary Tables S1 and S3.

### 2.6. Prioritised Candidate Panel for Orthogonal Validation

Convergent markers were assembled into a prioritised validation panel (Table 4). Rather than designing a separate EOPE-versus-LOPE panel—which the discovery analysis indicates would have no robust target—we propose a single multiplex measured in all three groups (HP, EOPE, LOPE). This design yields the two confirmatory disease-versus-healthy comparisons directly, while the EOPE-versus-LOPE contrast falls out as a built-in secondary test of subtype separation on the same measurements, without additional assays. The panel comprises the confirmatory disease markers (fibronectin, complement factor D, histidine-rich glycoprotein, glycoprotein hormones alpha chain, apolipoprotein E) plus two divergence-axis proteins (α1-antitrypsin and serum amyloid A-1) that capture the largest, opposite-direction differences between the subtype signatures. Under this design, a negative EOPE-versus-LOPE result would corroborate the molecular-continuum interpretation, whereas a positive result would reveal a subtype difference below the resolution of small-sample discovery proteomics; both outcomes are informative.

### 2.7. Independent ELISA Validation

The independent validation cohort comprised 29 women with early-onset preeclampsia, 29 with late-onset preeclampsia and 29 normotensive controls. The groups differed as expected in gestational age at diagnosis (median 30 versus 36 weeks), gestational age at delivery and birth weight, and in the frequency of magnesium sulfate use, confirming the intended clinical separation of the onset subtypes (Table 5).

**Figure 6:**
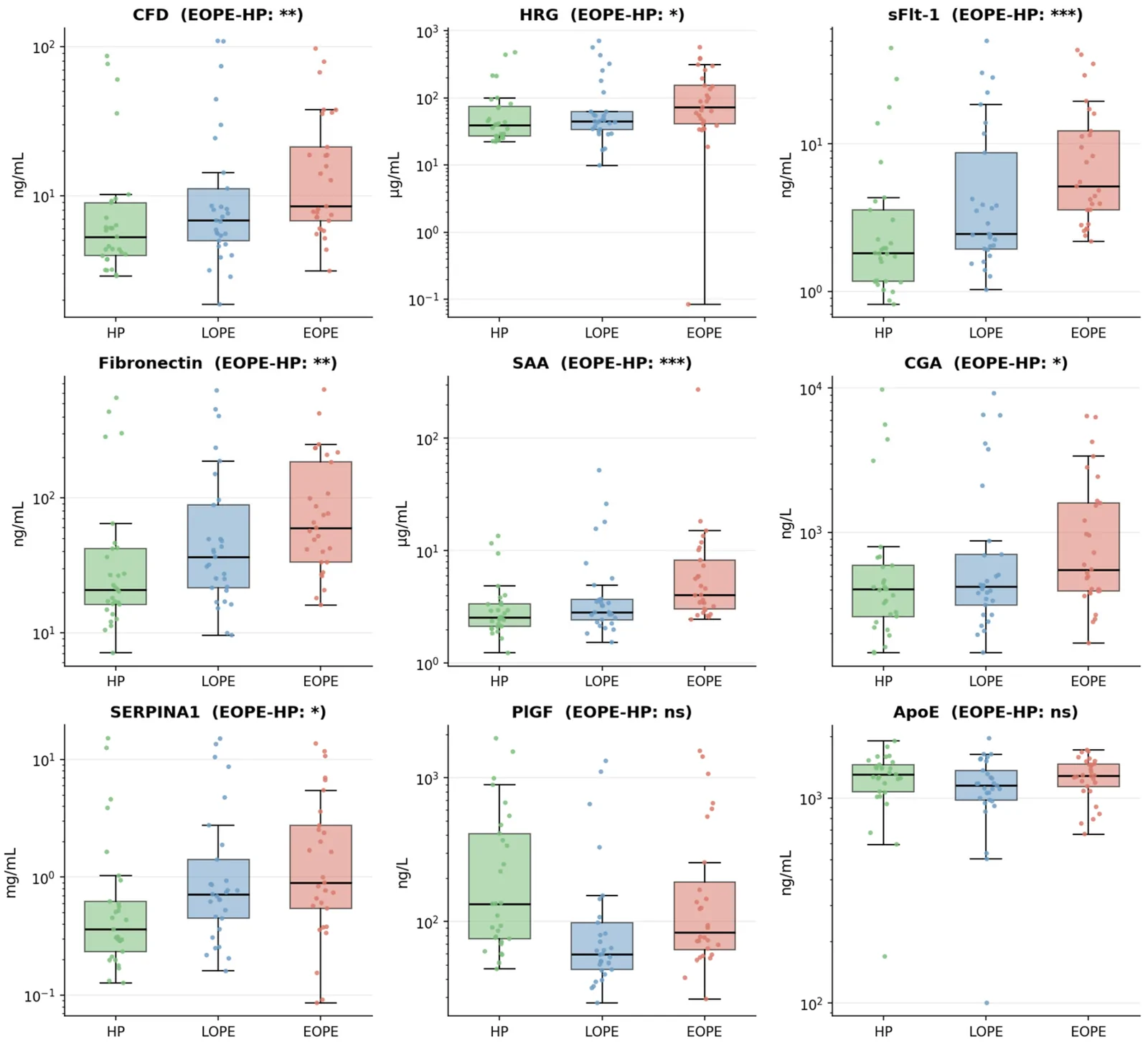
Distribution of ELISA-measured concentrations by group (healthy pregnancy, LOPE, EOPE) for the nine analytes (log scale; boxes show median and interquartile range, points show individual samples). Significance annotations refer to EOPE versus healthy pregnancy after Benjamini–Hochberg correction (*** q < 0.001, ** q < 0.01, * q < 0.05, ns not significant).

**Figure 7:**
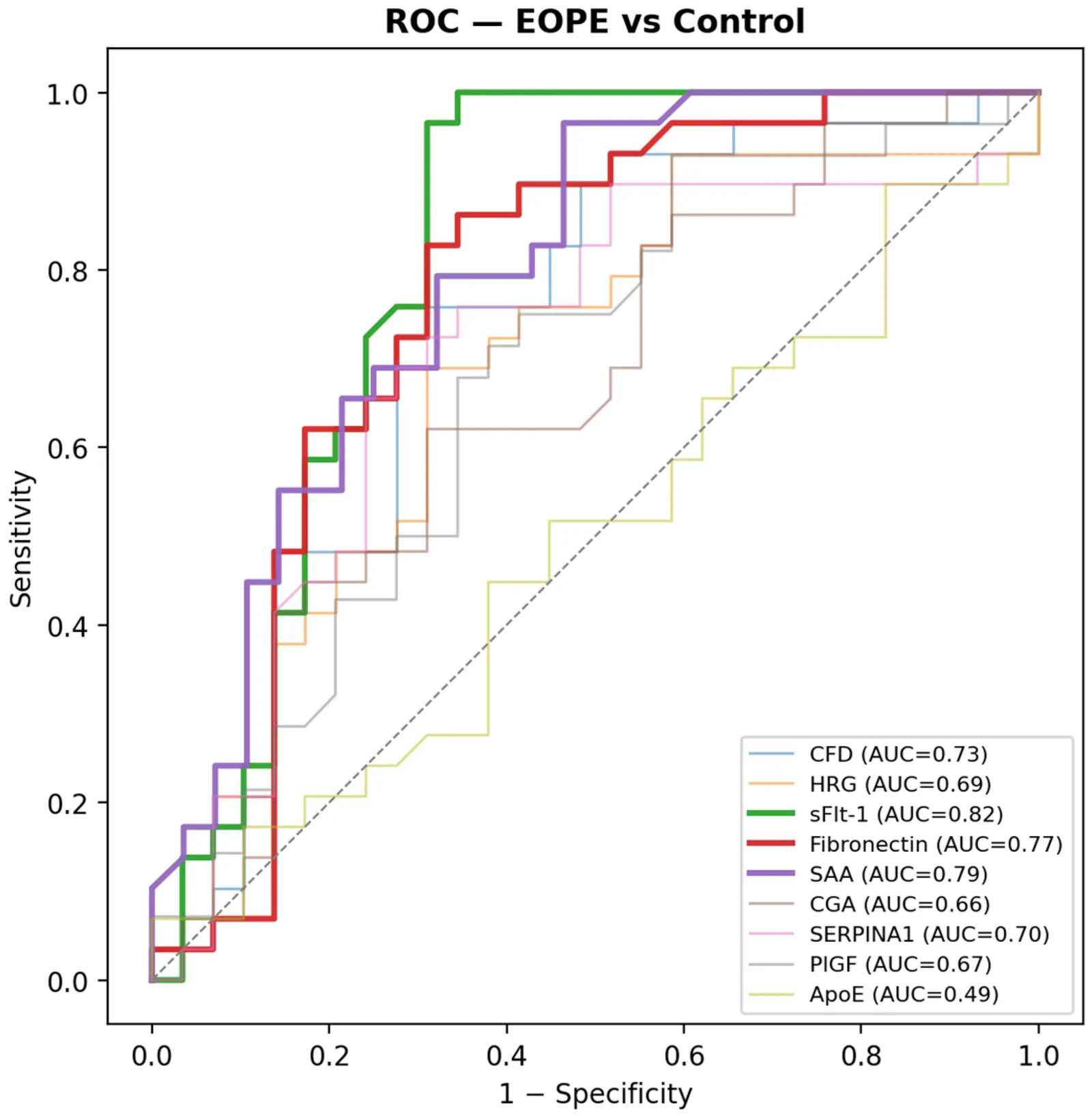
Receiver-operating-characteristic curves for discriminating EOPE from healthy pregnancy for each analyte, with areas under the curve. sFlt-1, serum amyloid A and fibronectin are the strongest discriminators; apolipoprotein E performs at chance.

The prioritised panel was measured by ELISA in an independent cohort of 87 women (29 EOPE, 29 LOPE, 29 healthy pregnancies), with each analyte quantified against a four-parameter logistic standard curve (R^2^ = 0.980–0.998). Early-onset preeclampsia was clearly separated from healthy pregnancy: eight of the nine measured proteins differed significantly after Benjamini–Hochberg correction (Table 6, Figure 6), with the largest effects for soluble fms-like tyrosine kinase-1 (sFlt-1; AUC 0.82), serum amyloid A (0.79) and fibronectin (0.77), followed by complement factor D, histidine-rich glycoprotein, α1-antitrypsin, glycoprotein hormones alpha chain and placental growth factor (AUC 0.66–0.73; all q < 0.05; Figure 7). Most discovery-derived candidate markers showed directionally concordant changes in the independent ELISA cohort, and the concordant elevation of sFlt-1—a clinically established, disease-elevated marker included as a directional control—supports the internal validity of the analysis.

Late-onset preeclampsia was more weakly distinguished from healthy pregnancy: only sFlt-1 (AUC 0.71), α1-antitrypsin (0.72) and placental growth factor (0.75) reached significance after correction, while the remaining markers showed concordant but non-significant trends—consistent with the milder, later-presenting phenotype and the limited power of a 29-per-group comparison.

In direct agreement with the discovery analysis, no protein discriminated EOPE from LOPE after multiple-testing correction (all q > 0.15), consistent with substantial proteomic overlap between the two onset subtypes in a separate cohort.

Of the nine analytes, apolipoprotein E did not distinguish any group (area under the curve ≈ 0.49 for early-onset versus healthy pregnancy) and did not reproduce its discovery-stage, late-onset-leaning signal—a non-validation most plausibly reflecting the marked between-individual variance of this high-abundance, lipoprotein-associated protein (shaped by lipid metabolism and the common APOE ε2/ε3/ε4 genotype), the competitive assay’s measurement of total rather than a specific proteoform, and the modest discovery effect (log2 fold-change ≈ 0.89), whereas placental growth factor was lower in both preeclampsia groups than in healthy pregnancy, reaching significance for late-onset disease (Table 6).

## 3. Discussion

This re-analysis produced four results. At the protein level, preeclampsia was clearly distinguished from normotensive pregnancy, and the disease-versus-healthy signatures agreed across differential testing, feature-selection stability and two complementary explanation methods. Over-representation analysis placed these signatures in coherent biological context—complement and coagulation for early-onset disease, lipoprotein and cholesterol pathways for late-onset disease. The two onset subtypes, by contrast, were not distinguishable from one another by any univariate or multivariate approach, in the discovery cohort or in an independent ELISA cohort. Finally, the prioritised panel received partial, direction-aware support when measured by ELISA, anchored by the expected behaviour of soluble fms-like tyrosine kinase-1 (sFlt-1).

The early-onset signature was dominated by complement, acute-phase and coagulation proteins—complement factor D, complement C1r, coagulation factor V, α2-macroglobulin, histidine-rich glycoprotein and inter-α-trypsin inhibitor heavy chain—and the enrichment analysis reinforced this reading, with KEGG complement and coagulation cascades, Reactome hemostasis and complement, and the Hallmark coagulation and complement sets all over-represented. This profile matches the placental-ischaemic, inflammatory stage-1 processes of the two-stage model [3,4] and accords closely with an independent proteomic analysis that identified complement and coagulation activation as the principal pathway in early-onset preeclampsia [11]. Recent proteomic surveys of early-onset disease—in serum [12], placental tissue [13] and circulating placenta-derived extracellular vesicles [14]—have likewise converged on inflammatory, complement and coagulation themes, which lends external plausibility to the signature recovered here despite the modest discovery sample.

The late-onset signature leaned instead toward apolipoproteins and lipid handling—apolipoprotein E, apolipoprotein C-IV and the APOC4–APOC2 locus—and the enrichment analysis returned Reactome plasma-lipoprotein assembly, remodelling and clearance, chylomicron assembly and liver-X-receptor cholesterol signalling, together with KEGG cholesterol metabolism. This is in keeping with the maternal metabolic and vascular background more often implicated in late-onset disease [1]. That late-onset preeclampsia was separated from controls less sharply than early-onset disease, both in classification and by ELISA, is consistent with its milder, later-presenting phenotype; the difficulty of the late-onset problem is underscored by a recent effort that quantified roughly seven thousand proteins to nominate new late-onset candidates beyond the angiogenic markers [15,16].

Fibronectin and complement factor D were shared across both disease-versus-healthy contrasts, pointing to a common endothelial and complement component that does not depend on onset timing. Any pathway-level reading of a few dozen differentially abundant proteins is necessarily provisional, and the enrichment—computed on small differentially abundant sets against a genome background—should be treated as descriptive support rather than as an independent quantitative result.

The clearest message of the study is a negative one. Neither differential testing nor leakage-free classification separated early- from late-onset disease; the classifiers that discriminated disease from health collapsed to chance for the subtype contrast (area under the curve ≈ 0.53–0.55), and their explanations were dominated by immunoglobulin variability rather than coherent biology. Because a multivariate model can recover structure that marginal tests miss, the coincidence of a univariate and a multivariate null is more informative than either alone, and it held under both feature-selection strategies and in the independent ELISA cohort. This fits the still-unresolved status of the early- versus late-onset distinction [3,17] and tempers reports that isolate individual discriminating proteins between the subtypes, which are hard to sustain in samples of this size [8].

Several explanations are compatible with this overlap. Early- and late-onset preeclampsia may converge on a shared final common pathway of endothelial dysfunction that dominates the circulating proteome even where upstream placental biology differs; the subtypes may lie along a severity continuum rather than in discrete molecular states; or subtype-specific differences may be confined to the placenta and its immediate interface, where single-cell and tissue studies do resolve cell-type-specific angiogenic and inflammatory signatures [13,17,18], and may be diluted below detection in peripheral blood. Our data cannot adjudicate between these possibilities, but they set a useful bound: in the maternal circulation of this cohort, the two onset subtypes do not carry a large, reproducible proteomic difference.

This should be distinguished from the growing literature on subtype prediction. Models that forecast which women will develop early- or late-onset disease, or that stratify risk within a subtype, combine clinical, ultrasound and angiogenic inputs and can perform well [19,20,21]; the present question is different—whether, once the subtypes are established, they are separable from each other by the circulating proteome alone. A model can predict the occurrence of a subtype without there being a clean molecular boundary between subtypes, and our results point to exactly this asymmetry.

The angiogenic axis remains the reference point for any circulating marker of preeclampsia. In the ELISA cohort, sFlt-1 was the strongest single discriminator of early-onset disease (area under the curve 0.82), consistent with its established elevation in preeclampsia and with the prognostic and predictive value of the sFlt-1/PlGF ratio [5,22,23,24]. This ratio is, however, a disease-versus-healthy tool rather than a subtype discriminator, and its clinical role is not displaced by anything reported here.

Methodologically, confining missing-value filtering, imputation, variance filtering, feature selection, class balancing and hyperparameter search to the training partition of each outer fold guards against the leakage that inflates performance in small-sample machine-learning studies [9]; in a sensitivity analysis, moving the label-independent preprocessing inside the folds lowered the disease-versus-healthy area under the curve only modestly and left the subtype null unchanged. Because plasma proteomic signatures of preeclampsia shift with cohort, demographics and sampling time [25], we treated agreement across independent lines of evidence—differential testing, selection stability and two explanation methods—as more trustworthy than any single model, and we report the strong disease-versus-healthy result and the null subtype result with equal weight.

In the independent validation cohort, whose early- and late-onset groups differed as expected in gestational age at diagnosis, gestational age at delivery and birth weight (Table 5), most discovery-derived candidate markers showed directionally concordant changes by ELISA: early-onset preeclampsia differed significantly from normotensive pregnancy for seven of nine analytes after correction, led by sFlt-1 (Table 6, Figure 6 and Figure 7). These findings provide partial independent support for the discovery-stage signatures in separately collected samples. Late-onset disease was separated from controls for fewer proteins, and the subtype contrast was again non-significant for every protein, reproducing the discovery-stage null.

One marker did not validate. Apolipoprotein E discriminated no group and did not reproduce its discovery-stage, late-onset leaning, most plausibly reflecting the modest discovery effect size and assay- or cohort-related differences. Placental growth factor, by contrast, decreased in disease as expected—significantly in late-onset disease—consistent with the physiological fall of circulating placental growth factor in preeclampsia [5] and complementing the rise in soluble fms-like tyrosine kinase-1 as the two arms of the angiogenic imbalance. This is reported without adjustment.

Several limitations qualify these conclusions. The discovery analysis re-uses a single, modestly sized dataset [8], and the signatures still warrant confirmation in larger, independently collected cohorts. The subtype groups are the smallest and most imbalanced, which limits power for the comparison of greatest interest. In the validation cohort, ELISA concentrations were measured in singlicate to keep all groups on one plate per protein. Gestational age at sampling could not be uniformly ascertained across all validation groups and therefore could not be incorporated as a covariate. The early-onset group also differed from the others in smoking prevalence, an imbalance that could influence some analytes and that a larger, matched design would address. Given the small per-group numbers and the sparse cells in some clinical comparisons, exact tests are preferable to asymptotic ones. For these reasons the panel should be read as hypothesis-generating rather than clinically actionable; the sFlt-1/PlGF ratio remains the validated clinical tool [5], and nothing reported here displaces it.

## 4. Materials and Methods

**Figure 8:**
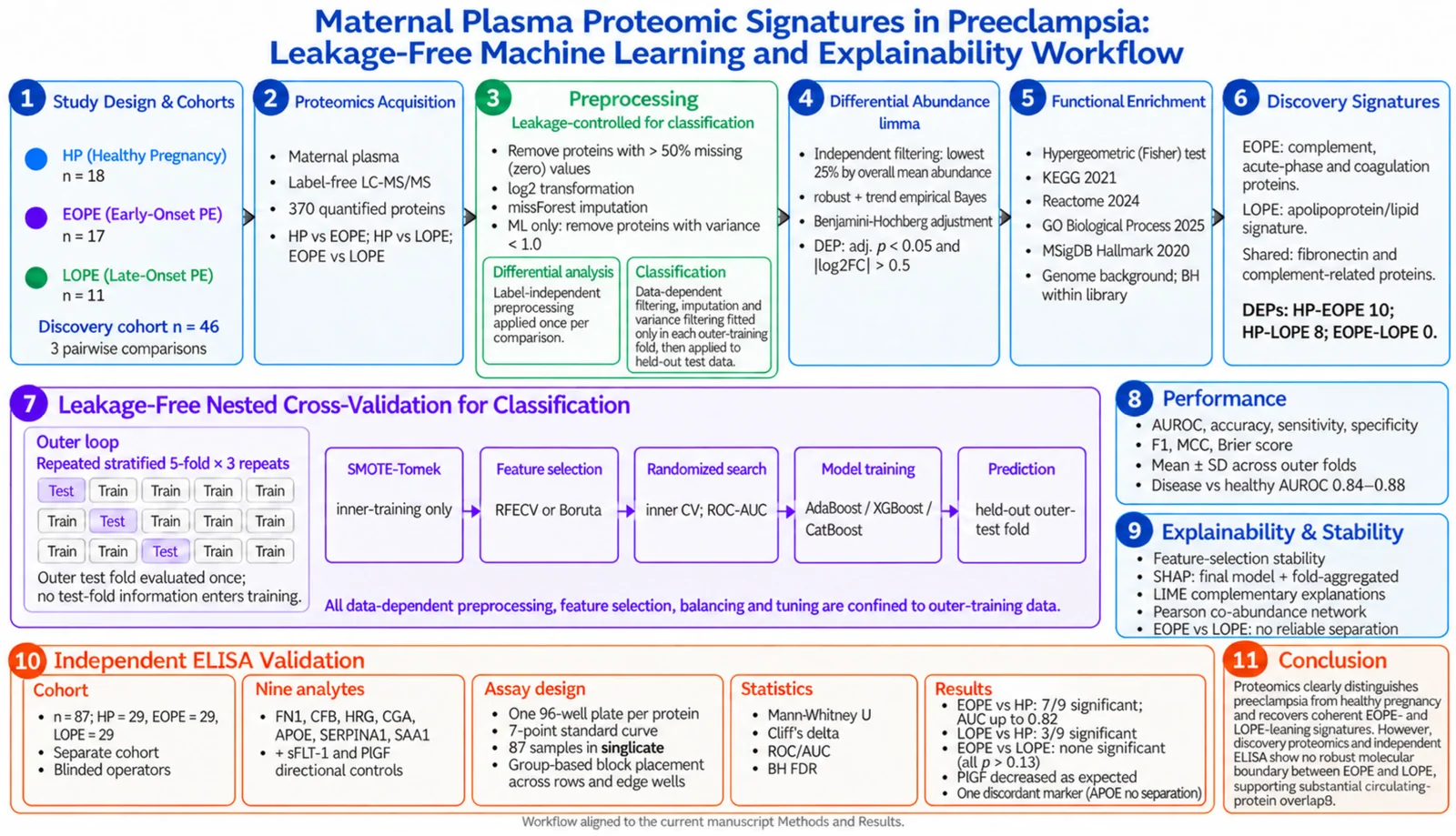
Overview of the study design and the leakage-free analytical workflow. HP, healthy pregnancy; EOPE and LOPE, early-onset and late-onset preeclampsia; DEP, differentially abundant protein. Plasma proteomes from the discovery cohort (healthy pregnancy, early-onset and late-onset preeclampsia) were analysed with all preprocessing and model-selection steps confined to the training partition of each cross-validation fold. Differential abundance (limma), functional over-representation, leakage-free nested cross-validation, and model explainability (SHAP, LIME, feature-selection stability) were followed by independent enzyme-linked immunosorbent assay validation of a nine-protein panel in a separate cohort. Abbreviations are as defined in the main text.

The overall study design and the leakage-free analytical workflow are summarised in Figure 8.

### 4.1. Cohort and Proteomic Data

Maternal plasma proteomes were obtained from 46 pregnant women: healthy pregnancy (HP, *n* = 18), EOPE (*n* = 17) and LOPE (*n* = 11). Preeclampsia was diagnosed by standard ACOG criteria, with EOPE and LOPE defined by onset before and from 34 weeks of gestation, respectively. Relative protein abundances were quantified by label-free LC–MS/MS, yielding 370 proteins quantified across samples. Analyses were performed for three pairwise comparisons: HP vs. EOPE, HP vs. LOPE, and EOPE vs. LOPE.

### 4.2. Preprocessing

Preprocessing used only label-independent operations: proteins with more than 50% missing (zero) values were removed, the remaining values were log2-transformed, missing values were imputed by missForest [26] (random-forest imputation), and, for the machine-learning analyses, proteins with variance below 1.0 were removed to limit uninformative features. For the differential analysis these steps were applied once per comparison. For the classification analysis, however, the data-dependent steps—missing-value filtering, imputation and variance filtering—were fit only on the outer-training partition of each cross-validation fold and then applied to the held-out test partition, so that no information from the test data entered any preprocessing step (Section 4.4).

### 4.3. Differential Abundance and Functional Enrichment

Differential abundance was assessed in R with limma (verison 3.68.5) [10]. Because the gestational-age covariate available in some prior studies was not available here, no covariate adjustment was applied, because participant-level body mass index and parity were likewise unavailable in the re-analysed discovery dataset and gestational age at sampling was not uniformly recorded across the groups; this is acknowledged as a limitation. A two-one-sided-test equivalence analysis of the early-versus-late contrast found only 38% of proteins statistically equivalent at a margin of |log2 fold-change| below 1.0 and 59% inconclusive, so true molecular overlap cannot be affirmed over insufficient power; consistently, the discovery contrasts reached 80% power only for large effects (early-onset versus healthy Cohen d at least 0.98, late-onset versus healthy at least 1.11) and the validation for d at least 0.75. Once apolipoprotein E failed to validate—and indeed trended in the opposite direction in late-onset disease (area under the curve 0.39)—no validation-panel analyte was specific to a single subtype: the validated markers were elevated in both subtypes, more strongly in early-onset disease, so the discovery-stage subtype-leaning associations denote directions of enrichment rather than validated subtype-specific biomarkers, and the independent data support a shared, severity-graded signature. Adjustment for age and body mass index did not alter the null apolipoprotein E result (adjusted q = 0.85), and APOE ε2/ε3/ε4 genotype, a major determinant of circulating apolipoprotein E, was unavailable for both cohorts. A per-participant soluble fms-like tyrosine kinase-1 to placental growth factor ratio could not be derived because the two analytes were assayed on separate plates with differing layouts and no linking identifier. Lower limits of detection and quantification and intra- and inter-assay coefficients of variation are provided in Supplementary Table S5; independent dilutional-linearity and spike-and-recovery data were not generated, and suboptimal analyte-specific performance cannot be excluded as a contributor to the apolipoprotein E non-validation. To increase power and robustness in this small-sample setting we used (i) independent filtering—removal, before testing, of the lowest 25% of proteins by overall mean abundance, a criterion independent of group label—and (ii) robust, trended empirical Bayes moderation (eBayes with trend = TRUE, robust = TRUE), which down-weights hyper-variable features such as immunoglobulin variable regions. Moderated-t *p*-values were Benjamini–Hochberg adjusted within each pairwise comparison; a more conservative false-discovery-rate correction pooled across all three comparisons (757 tests) reduced the disease-versus-healthy signatures (early-onset from 10 to 5 and late-onset from 8 to 3 proteins) but preserved the leading markers (complement factor D, fibronectin, histidine-rich glycoprotein, alpha-2-macroglobulin and complement C1r) and the null early-versus-late result. Differentially abundant proteins (DEPs) were defined at the confirmatory threshold adjusted *p* < 0.05 and |log2FC| > 0.5; a discovery threshold (adjusted *p* < 0.10, |log2FC| > 0.5) was reported in parallel for all three comparisons.

Functional over-representation of the differentially abundant proteins (Benjamini–Hochberg-adjusted *p* < 0.05, |log2 fold change| > 0.5) was assessed for each disease-versus-healthy comparison with the hypergeometric (Fisher) test against the KEGG 2021, Reactome 2024, Gene Ontology Biological Process 2025 and MSigDB Hallmark 2020 gene-set libraries, using a genome background; enrichment *p*-values were Benjamini–Hochberg-adjusted within each library. The early- versus late-onset comparison yielded no differentially abundant proteins and was not tested. Enriched terms are reported in Supplementary Table S2.

### 4.4. Classification by Nested Cross-Validation

Classification used a fully nested, leakage-free cross-validation. Within each outer fold of a repeated stratified k-fold scheme (5 folds × 3 repeats), all data-dependent steps—missing-value filtering, missForest imputation, variance filtering, feature selection, class balancing and hyperparameter search—were fit exclusively on the outer-training partition and applied to the held-out outer-test partition, which was evaluated once. Feature selection used recursive feature elimination with cross-validation (RFECV, random-forest estimator) and the Boruta [27] algorithm, reported separately. Class imbalance was addressed with SMOTE [28]-Tomek embedded inside the modelling pipeline so that synthetic samples were generated only within inner-training folds; the number of synthetic neighbours was adapted to the minority count to remain valid in small folds. Three classifiers—AdaBoost, XGBoost [29] and CatBoost [30]—were each tuned by randomised search (inner cross-validation, ROC-AUC) within every outer fold and reported separately, so that model-family choice is read from, rather than hidden within, the results. Performance metrics (AUROC, accuracy, sensitivity, specificity, F1, Matthews correlation coefficient [MCC], Brier score) were aggregated across outer folds as mean ± standard deviation.

### 4.5. Selection Stability, Explainability, and Software

Feature-selection stability was quantified as the fraction of outer folds in which each protein was selected. Model explanations used SHAP [31] by two methods. Method 1 (final model) fit a single model on all samples after one feature-selection pass and is reported as the primary, interpretable importance ranking; it is descriptive and carries no performance claim. Method 2 (fold-aggregated) computed SHAP on each outer-test partition using that fold’s own model and aggregated the results, mirroring the leakage-free evaluation. XGBoost and CatBoost models were explained with TreeExplainer, and AdaBoost with the model-agnostic permutation explainer applied to predicted probabilities. As a complementary cross-check, LIME [32] (local interpretable model-agnostic explanations) was applied to the final model and aggregated as the mean absolute local weight across participants. Co-abundance structure among the leading convergent proteins was summarised by pairwise Pearson correlation across the whole cohort and visualised as a circular network. Differential analysis was performed in R version 4.6.1 (R Foundation for Statistical Computing, Vienna, Austria) using limma 3.68.4 (Bioconductor 3.23), with missing values imputed by the missForest package 1.6.1 and volcano plots drawn with ggplot2 4.0.3 and ggrepel 0.9.7. Preprocessing, enrichment, nested cross-validation, feature selection, class balancing and SHAP explanations were run in Python 3.14.5 (Python Software Foundation, Wilmington, DE, USA) using scikit-learn 1.9.1 (including AdaBoostClassifier), imbalanced-learn 0.14.2, XGBoost 3.4.1, CatBoost 1.2.10, BorutaPy 0.4.3 and SHAP 0.52.0. LIME, co-abundance, power, equivalence, DeLong and ELISA analyses were performed in Python 3.12.3 using NumPy 2.4.4, pandas 3.0.2, SciPy 1.17.1, scikit-learn 1.8.0, statsmodels 0.15.0, SHAP 0.52.0 and LIME 0.2.0.1.

### 4.6. Clinical Interpretation of Independent ELISA Validation Findings

The convergent candidate proteins were validated by enzyme-linked immunosorbent assay (ELISA) in an independent cohort, collected separately from the discovery samples (results in Section 2.7). Three groups—healthy pregnancy, EOPE and LOPE—were enrolled with 29 participants per group (total *n* = 87). Participants were recruited at a tertiary obstetrics unit, and preeclampsia was diagnosed and classified into early- and late-onset forms by attending obstetricians according to standard clinical criteria; baseline demographic, obstetric and clinical characteristics are summarised in Table 5. Each candidate protein was quantified with a dedicated commercial ELISA kit (Bioassay Technology Laboratory, Shanghai, China; catalogue numbers in Supplementary Table S5); nine analytes were measured, comprising the convergent candidates defined a priori as the proteins with concordant support across limma differential abundance, nested cross-validation feature-selection stability and SHAP importance in the disease-versus-healthy contrasts (fibronectin, complement factor D, histidine-rich glycoprotein, glycoprotein hormones alpha chain, apolipoprotein E, α1-antitrypsin and serum amyloid A-1) together with soluble fms-like tyrosine kinase-1 (sFlt-1) and placental growth factor (PlGF) as clinically established directional controls. Absorbance was read at 450 nm on an Epoch microplate spectrophotometer (BioTek Instruments, Winooski, VT, USA); assay ranges, sensitivities and coefficients of variation are provided in Supplementary Table S5. To eliminate plate-to-plate (batch) effects from the group comparison, all three groups were assayed together on a single 96-well plate per protein: a seven-point standard curve occupied the first column and the 87 samples filled the remaining wells, measured in singlicate. Samples were loaded group-wise in contiguous well blocks, with all three groups assayed together on the same plate per analyte under identical conditions, and operators were blinded to group allocation. Gestational age at sampling, maternal age and body mass index were recorded. Gestational age at sampling was not available as a covariate in the discovery analysis and could not be uniformly recorded across groups in the validation cohort.

Disease-versus-healthy markers were compared between groups by Mann–Whitney U tests with effect sizes (Cliff’s delta) and receiver-operating-characteristic analysis (AUC), with Benjamini–Hochberg control of the false-discovery rate across proteins. Because the discovery analysis indicated no separable EOPE–LOPE proteome, the EOPE-versus-LOPE comparison was treated as a secondary, power-limited test. With 29 participants per group and singlicate measurement, this comparison is powered to detect only large between-subtype differences; accordingly, a non-significant result is interpreted as the absence of a detectable large effect rather than as proof of identity. Single-well (singlicate) measurement, adopted to keep all groups on one plate per protein, is acknowledged as a limitation; because samples were arranged group-wise in contiguous blocks rather than randomised across the plate, positional or edge effects cannot be excluded as a contributor to between-group differences, and samples were not measured in replicate, so the intra-assay coefficient of variation could not be estimated. The validation was conducted under separate institutional ethics approval with written informed consent. The validation cohort was approved by the İnönü University Scientific Research and Publication Ethics Committee (Health Sciences) on 10 March 2026 (session 5, decision number 2026/6666).

## 5. Conclusions

In maternal plasma, leakage-free proteomic analysis distinguishes preeclampsia from healthy pregnancy and recovers convergent, biologically coherent EOPE- and LOPE-leaning signatures, yet provides little evidence for a sharp circulating-proteome boundary between the two subtypes—consistent with substantial molecular overlap between the subtypes. A convergence-based candidate panel underwent preliminary orthogonal validation by ELISA and warrants further assessment in larger, clinically matched cohorts.

## Figures and Tables

**Table 1:** Cohort composition and pairwise-comparison overview. DEPs, differentially abundant proteins (limma, adjusted *p* < 0.05 and |log2FC| > 0.5).

Group/Comparison	*n*	DEPs (adj.*p* < 0.05)	Description
Healthy pregnancy (HP)	18	-	Normotensive control
Early-onset PE (EOPE)	17	-	Onset < 34 weeks
Late-onset PE (LOPE)	11	-	Onset ≥ 34 weeks
HP vs. EOPE	35	11	Disease vs. healthy (early)
HP vs. LOPE	29	8	Disease vs. healthy (late)
EOPE vs. LOPE	28	0	Subtype vs. subtype

**Table 2:** Representative differentially abundant proteins (selection of the 8 + 11 DEPs). All up-regulated versus healthy pregnancy. Full lists are provided in Supplementary Table S4.

Protein (Gene)	Comparison	log2FC	adj.*p*	Axis
Fibronectin (*FN1*)	HP vs. LOPE	+1.43	0.006	Endothelial (shared)
Apolipoprotein E (*APOE*)	HP vs. LOPE	+0.89	0.028	Lipid (LOPE)
APOC4-APOC2 readthrough	HP vs. LOPE	+0.92	0.036	Lipid (LOPE)
Apolipoprotein C-IV (*APOC4*)	HP vs. LOPE	+0.92	0.036	Lipid (LOPE)
Complement C1r (*C1R*)	HP vs. LOPE	+0.52	0.036	Complement (shared)
Complement factor D (*CFD*)	HP vs. EOPE	+1.60	0.018	Complement (shared)
Histidine-rich glycoprotein (*HRG*)	HP vs. EOPE	+1.25	0.010	Coagulation (EOPE)
Fibronectin (*FN1*)	HP vs. EOPE	+1.17	0.010	Endothelial (shared)
α2-macroglobulin (*A2M*)	HP vs. EOPE	+0.57	0.010	Acute-phase (EOPE)
Coagulation factor V (*F5*)	HP vs. EOPE	+0.62	0.042	Coagulation (EOPE)

**Table 3:** Nested cross-validation performance for the disease-versus-healthy comparisons (outer-fold mean ± SD). FS, feature selection. All three classifiers were evaluated in every outer fold; for readability the classifier with the highest mean out-of-fold AUROC is shown, and complete per-model results are provided in Supplementary Table S3. The displayed model is descriptive and was not used to select features, tune the pipeline or define the panel. The EOPE-versus-LOPE comparison is reported in Supplementary Table S1.

Comparison (FS)	Model	AUROC	Accuracy	F1	MCC	Brier
HP vs. EOPE (Boruta)	CatBoost	0.88 ± 0.12	0.79 ± 0.16	0.79 ± 0.16	0.61 ± 0.31	0.16 ± 0.05
HP vs. EOPE (RFECV)	CatBoost	0.89 ± 0.12	0.82 ± 0.11	0.83 ± 0.11	0.68 ± 0.20	0.18 ± 0.05
HP vs. LOPE (Boruta)	CatBoost	0.90 ± 0.10	0.80 ± 0.14	0.62 ± 0.35	0.54 ± 0.37	0.16 ± 0.04
HP vs. LOPE (RFECV)	XGBoost	0.88 ± 0.16	0.79 ± 0.14	0.63 ± 0.35	0.53 ± 0.36	0.15 ± 0.08

**Table 4:** Proposed single-multiplex validation panel, measured in all three groups (HP, EOPE, LOPE). The HP-versus-EOPE and HP-versus-LOPE comparisons are confirmatory; the EOPE-versus-LOPE comparison is a built-in secondary test of subtype separation. stab, nested-CV selection stability.

Protein (Gene)	Primary Role	Evidence	Rationale	Priority
Fibronectin (*FN1*)	LOPE marker	limma + stab + SHAP + LIME	Strongest, cross-cutting	1
Complement factor D (*CFD*)	EOPE + shared	limma + stab + SHAP + LIME	High-effect complement	1
Histidine-rich glycoprotein (*HRG*)	EOPE marker	limma + stab + SHAP + LIME	Coagulation/angiogenesis	1
Glycoprotein hormones α (*CGA*)	EOPE marker	stab + SHAP + LIME	ML-discovered placental	2
Apolipoprotein E (*APOE*)	LOPE marker	limma + stab	LOPE lipid axis	2
α1-antitrypsin (*SERPINA1*)	Subtype axis	divergence (exploratory)	Opposite direction (EOPE ↑/LOPE ↓)	3
Serum amyloid A-1 (*SAA1*)	Subtype axis	divergence (exploratory)	EOPE-skewed	3

Note: *↑* indicates an increase; *↓* indicates a decrease.

**Table 5:** Baseline characteristics of the independent ELISA validation cohort. Continuous variables are median [interquartile range] with Kruskal–Wallis tests; categorical variables are *n* (%) with the Freeman–Halton exact test (Monte-Carlo permutation, 20,000 permutations). GA, gestational age.

Characteristic	EOPE (*n* = 29)	LOPE (*n* = 29)	Control (*n* = 29)	*p*
**Demographics & obstetric history**				
Age (years)	33.0 [29.0–35.0]	32.0 [27.0–35.0]	30.0 [28.0–32.0]	0.125
Gravidity	2.0 [1.0–4.0]	2.0 [1.0–4.0]	2.0 [1.0–3.0]	0.522
Parity	1.0 [0.0–2.0]	0.0 [0.0–1.0]	0.0 [0.0–1.0]	0.046
Abortus	0.0 [0.0–1.0]	0.0 [0.0–1.0]	0.0 [0.0–1.0]	0.653
BMI (kg/m^2^)	29.0 [28.0–35.0]	32.0 [28.0–35.0]	29.0 [27.0–30.0]	0.079
Previous preeclampsia	1 (3%)	1 (3%)	0 (0%)	1.000
Previous caesarean	9 (31%)	9 (31%)	8 (28%)	1.000
**Comorbidities**				
Chronic hypertension	5 (17%)	3 (10%)	0 (0%)	0.090
Diabetes	4 (14%)	5 (17%)	1 (3%)	0.321
Renal disease	3 (10%)	0 (0%)	0 (0%)	0.110
Thyroid disease	2 (7%)	9 (31%)	4 (14%)	0.060
Autoimmune disease	3 (10%)	8 (28%)	5 (17%)	0.278
IVF pregnancy	2 (7%)	3 (10%)	1 (3%)	0.867
**Preeclampsia features**				
GA at diagnosis (wk)	30.0 [28.0–32.0]	36.0 [35.0–38.0]	—	<0.001
Proteinuria (mg)	795.0 [375.0–1562.0]	391.0 [336.0–897.0]	37.0 [0.0–152.0]	<0.001
Magnesium sulfate	14 (48%)	1 (3%)	0 (0%)	<0.001
HELLP syndrome	0 (0%)	2 (7%)	0 (0%)	0.326
Eclampsia	0 (0%)	0 (0%)	0 (0%)	—
Intensive care	0 (0%)	0 (0%)	0 (0%)	—
**Pregnancy outcomes**				
GA at delivery (wk)	34.0 [31.0–37.0]	37.0 [37.0–38.0]	39.0 [39.0–39.0]	<0.001
Birth weight (g)	2140.0 [1600.0–2590.0]	2880.0 [2560.0–3300.0]	3230.0 [3070.0–3516.0]	<0.001
Perinatal death	2 (7%)	0 (0%)	0 (0%)	0.327

**Table 6:** Independent ELISA validation of the prioritised panel. Placental growth factor decreased in disease (the inverse of the other markers); its area-under-the-curve values reflect discrimination by lower concentration. AUC (disease as positive class) and Benjamini–Hochberg-adjusted *p* (q) for each protein, for EOPE and LOPE versus healthy pregnancy (*n* = 29 per group). No protein separated EOPE from LOPE after correction (all q > 0.15).

Protein	AUC (EOPE–HP)	q (EOPE–HP)	AUC (LOPE–HP)	q (LOPE–HP)
CFD	0.73	0.005	0.61	0.24
HRG	0.69	0.022	0.59	0.26
sFlt-1	0.82	<0.001	0.71	0.022
Fibronectin	0.77	0.001	0.65	0.13
SAA	0.79	<0.001	0.60	0.24
CGA	0.66	0.048	0.56	0.43
SERPINA1	0.70	0.015	0.72	0.016
PlGF	0.59	0.032	0.75	0.011
ApoE	0.49	0.93	0.39	0.24

## Data Availability

The raw data supporting the conclusions of this article will be made available by the authors on request.

## Supplementary Materials

The following supporting information can be downloaded at: https://www.mdpi.com/article/10.3390/ijms27209275/s1.
