# Supplementary material for: Maternal Plasma Proteomic Signatures Distinguish Preeclampsia from Healthy Pregnancy but Not Early-Onset from Late-Onset Subtypes: A Leakage-Free Machine-Learning and Explainability Study
Source: Int J Mol Sci. 2026 Oct 19;27(20):9275. doi: 10.3390/ijms27209275 (PMC13704937; doi:10.3390/ijms27209275)
Supplement: Supplementary file 1 [file ijms-27-09275-s001.zip › ijms-4559286-supplementary.pdf]

Supplementary Materials

Maternal plasma proteomic signatures distinguish preeclampsia from healthy pregnancy but not early-onset from late-onset subtypes: a leakage-free machine-learning and explainability study

Supplementary Table S1. Nested cross-validation performance for the early-onset versus late-onset comparison (leakage-free pipeline; 5×3 repeated stratified outer folds). Values are mean±SD across outer folds.

Comparison	FS	Best model	AUROC	Accuracy	Sensitivity	Specificity	F1	MCC	Brier
EOPE-LOPE	RFECV	AdaBoost	0.551±0.305	0.636±0.208	0.444±0.381	0.743±0.249	0.440±0.345	0.216±0.467	0.244±0.057
EOPE-LOPE	BORUTA	XGBoost	0.528±0.318	0.611±0.201	0.422±0.364	0.728±0.235	0.411±0.328	0.160±0.451	0.283±0.122

Supplementary Table S2. Functional over-representation of the differentially abundant proteins (hypergeometric test, Benjamini–Hochberg FDR per library, genome background). Significant terms (FDR<0.05) shown. k/K, overlap over gene-set size.

Comparison	Library	Term	k/K	FDR	Genes
EOPE vs Control	KEGG 2021	Complement and coagulation cascades	4/85	<0.001	A2M, C1R, CFD, F5
EOPE vs Control	KEGG 2021	Staphylococcus aureus infection	2/95	0.009	C1R, CFD
EOPE vs Control	KEGG 2021	Coronavirus disease	2/232	0.034	C1R, CFD
EOPE vs Control	Reactome 2024	Platelet Degranulation	6/128	<0.001	A2M, CFD, F5, FN1, HRG, ITIH3
EOPE vs Control	Reactome 2024	Response to Elevated Platelet Cytosolic Ca2+	6/133	<0.001	A2M, CFD, F5, FN1, HRG, ITIH3
EOPE vs Control	Reactome 2024	Platelet Activation, Signaling and Aggregation	6/262	<0.001	A2M, CFD, F5, FN1, HRG, ITIH3
EOPE vs Control	Reactome 2024	Hemostasis	6/707	<0.001	A2M, CFD, F5, FN1, HRG, ITIH3
EOPE vs Control	Reactome 2024	Complement Cascade	3/139	<0.001	C1R, CFD, CPN2
EOPE vs Control	Reactome 2024	Formation of Fibrin Clot (Clotting Cascade)	2/39	0.002	A2M, F5
EOPE vs Control	Reactome 2024	Initial Triggering of Complement	2/104	0.013	C1R, CFD
EOPE vs Control	Reactome 2024	Post-translational Protein Phosphorylation	2/107	0.013	F5, FN1
EOPE vs Control	Reactome 2024	Regulation of IGF Transport and Uptake by Insulin-like Growth Factor Binding Proteins (IGFBPs)	2/124	0.014	F5, FN1
EOPE vs Control	Reactome 2024	Regulation of Complement Cascade	2/128	0.014	C1R, CPN2
EOPE vs Control	GO Biological Process 2025	Hemoglobin Metabolic Process (GO:0020027)	1/5	0.045	AMBP
EOPE vs Control	GO Biological Process 2025	Humoral Immune Response Mediated by Circulating Immunoglobulin (GO:0002455)	1/6	0.045	C1R
EOPE vs Control	GO Biological Process 2025	Integrin Activation (GO:0033622)	1/8	0.045	FN1
EOPE vs Control	GO Biological Process 2025	Negative Regulation of Transforming Growth Factor Beta Production (GO:0071635)	1/8	0.045	FN1
EOPE vs Control	GO Biological Process 2025	Heme Catabolic Process (GO:0042167)	1/10	0.045	AMBP
EOPE vs Control	GO Biological Process 2025	Pigment Catabolic Process (GO:0046149)	1/10	0.045	AMBP
EOPE vs Control	GO Biological Process 2025	Porphyrin-Containing Compound Catabolic Process (GO:0006787)	1/12	0.045	AMBP
EOPE vs Control	GO Biological Process 2025	Regulation of Transforming Growth Factor Beta Production (GO:0071634)	1/12	0.045	FN1
EOPE vs Control	MSigDB Hallmark 2020	Coagulation	5/138	<0.001	A2M, C1R, CFD, FN1, HRG
EOPE vs Control	MSigDB Hallmark 2020	Complement	3/200	<0.001	C1R, F5, FN1
LOPE vs Control	KEGG 2021	Cholesterol metabolism	2/50	0.003	APOC2, APOE
LOPE vs Control	KEGG 2021	Complement and coagulation cascades	2/85	0.005	A2M, C1R
LOPE vs Control	Reactome 2024	Plasma Lipoprotein Assembly	4/19	<0.001	A2M, APOC2, APOC4, APOE
LOPE vs Control	Reactome 2024	Plasma Lipoprotein Assembly, Remodeling, and Clearance	4/75	<0.001	A2M, APOC2, APOC4, APOE
LOPE vs Control	Reactome 2024	NR1H3 & NR1H2 Regulate Gene Expression Linked to Cholesterol Transport and Efflux	3/44	<0.001	APOC2, APOC4, APOE
LOPE vs Control	Reactome 2024	NR1H2 and NR1H3-mediated Signaling	3/54	<0.001	APOC2, APOC4, APOE
LOPE vs Control	Reactome 2024	Chylomicron Assembly	2/10	<0.001	APOC2, APOE
LOPE vs Control	Reactome 2024	Chylomicron Remodeling	2/10	<0.001	APOC2, APOE
LOPE vs Control	Reactome 2024	HDL Remodeling	2/10	<0.001	APOC2, APOE
LOPE vs Control	Reactome 2024	Regulation of Complement Cascade	3/128	<0.001	C1R, CPN1, IGLV7-46
LOPE vs Control	Reactome 2024	Complement Cascade	3/139	<0.001	C1R, CPN1, IGLV7-46
LOPE vs Control	Reactome 2024	Plasma Lipoprotein Remodeling	2/34	<0.001	APOC2, APOE
LOPE vs Control	GO Biological Process 2025	Acylglycerol Homeostasis (GO:0055090)	3/26	<0.001	APOC2, APOC4, APOE
LOPE vs Control	GO Biological Process 2025	Triglyceride Homeostasis (GO:0070328)	3/34	<0.001	APOC2, APOC4, APOE

Comparison	Library	Term	k/K	FDR	Genes
LOPE vs Control	GO Biological Process 2025	Very-Low-Density Lipoprotein Particle Remodeling (GO:0034372)	2/6	<0.001	APOC2, APOE
LOPE vs Control	GO Biological Process 2025	Triglyceride-Rich Lipoprotein Particle Remodeling (GO:0034370)	2/7	<0.001	APOC2, APOE
LOPE vs Control	GO Biological Process 2025	Negative Regulation of Cholesterol Transport (GO:0032375)	2/10	<0.001	APOC2, APOE
LOPE vs Control	GO Biological Process 2025	Phospholipid Efflux (GO:0033700)	2/12	<0.001	APOC2, APOE
LOPE vs Control	GO Biological Process 2025	Reverse Cholesterol Transport (GO:0043691)	2/16	<0.001	APOC2, APOE
LOPE vs Control	GO Biological Process 2025	Cholesterol Efflux (GO:0033344)	2/23	<0.001	APOC2, APOE
LOPE vs Control	GO Biological Process 2025	Negative Regulation of Lipid Metabolic Process (GO:0045833)	2/33	0.002	APOC2, APOE
LOPE vs Control	GO Biological Process 2025	Positive Regulation of Lipid Biosynthetic Process (GO:0046889)	2/40	0.002	APOC2, APOE
LOPE vs Control	MSigDB Hallmark 2020	Coagulation	5/138	<0.001	A2M, APOC2, C1R, CPN1, FN1
LOPE vs Control	MSigDB Hallmark 2020	Complement	2/200	0.009	C1R, FN1

Supplementary Table S3. Complete nested cross-validation performance for all comparisons and both feature-selection strategies (leakage-free pipeline).

Comparison	FS	Best model	AUROC	Accuracy	Sensitivity	Specificity	F1	MCC	Brier
HP-LOPE	RFECV	CatBoost	0.837±0.148	0.740±0.165	0.595±0.343	0.821±0.191	0.583±0.308	0.443±0.371	0.188±0.046
HP-LOPE	BORUTA	CatBoost	0.841±0.153	0.727±0.171	0.589±0.310	0.811±0.235	0.578±0.301	0.423±0.364	0.203±0.092
HP-EOPE	RFECV	CatBoost	0.883±0.152	0.790±0.126	0.856±0.165	0.739±0.215	0.801±0.115	0.613±0.253	0.180±0.046
HP-EOPE	BORUTA	CatBoost	0.878±0.161	0.752±0.122	0.817±0.207	0.700±0.172	0.758±0.121	0.546±0.238	0.174±0.049
EOPE-LOPE	RFECV	AdaBoost	0.551±0.305	0.636±0.208	0.444±0.381	0.743±0.249	0.440±0.345	0.216±0.467	0.244±0.057
EOPE-LOPE	BORUTA	XGBoost	0.528±0.318	0.611±0.201	0.422±0.364	0.728±0.235	0.411±0.328	0.160±0.451	0.283±0.122

Supplementary Table S4. Differentially abundant proteins for each disease-versus-healthy comparison (moderated-t, Benjamini–Hochberg-adjusted p<0.05 and |log2 fold change|>0.5). Positive log2FC indicates higher abundance in disease.

Protein	log2FC	Adjusted p	Regulation
**EOPE versus Control (n = 10)**			
Complement factor D	+1.60	1.76e-02	Up
Histidine rich glycoprotein	+1.25	1.03e-02	Up
Fibronectin	+1.17	1.03e-02	Up
Inter alpha trypsin inhibitor heavy chain H3	+0.93	3.64e-02	Up
Protein AMBP	+0.79	3.99e-02	Up
Coagulation factor V	+0.62	3.99e-02	Up
Carboxypeptidase N subunit 2	+0.62	3.99e-02	Up
Alpha 2 macroglobulin	+0.57	1.03e-02	Up
Complement C1r subcomponent	+0.51	1.39e-02	Up
Immunoglobulin lambda variable 3 9	-1.24	3.99e-02	Down
**LOPE versus Control (n = 8)**			
Immunoglobulin lambda variable 7 46	+1.46	4.42e-02	Up
Fibronectin	+1.43	6.08e-03	Up
APOC4 APOC2 readthrough NMD candidate	+0.92	3.59e-02	Up
Apolipoprotein C IV	+0.92	3.59e-02	Up
Apolipoprotein E	+0.89	2.79e-02	Up
Alpha 2 macroglobulin	+0.54	4.42e-02	Up
Complement C1r subcomponent	+0.52	3.57e-02	Up
Carboxypeptidase N catalytic chain	+0.52	4.42e-02	Up

Supplementary Table S5. Enzyme-linked immunosorbent assay kit and quality-control details. All analytes were measured with commercial ELISA kits (Bioassay Technology Laboratory, Shanghai, China); eight were sandwich assays and apolipoprotein E was a competitive assay. Catalogue number, standard-curve range, sensitivity (limit of detection) and intra- and inter-assay coefficients of variation are per the manufacturer inserts; lot numbers are recorded on each kit box. N>ULOQ / N<LLOQ, number of the 87 samples with back-calculated concentrations above the upper or below the lower limit of quantification.

Analyte	Manufacturer	Catalogue no.	Lot no.	Assay range	Sensitivity	Intra-assay CV	Inter-assay CV	N>ULOQ	N<LLOQ
CFD	BT-LAB	E0319Hu	—	0.5–100 ng/mL	0.21 ng/mL	<8%	<10%	2	0

Analyte	Manufacturer	Catalogue no.	Lot no.	Assay range	Sensitivity	Intra-assay CV	Inter-assay CV	N>ULOQ	N<LLOQ
HRG	BT-LAB	E1487Hu	—	3–900 µg/mL	1.44 µg/mL	<8%	<10%	0	1
sFlt-1	BT-LAB	E4049hu	—	0.1–40 ng/mL	0.04 ng/mL	<8%	<10%	4	0
Fibronectin	BT-LAB	E2002Hu	—	2–600 ng/mL	1.02 ng/mL	<8%	<10%	2	0
SAA	BT-LAB	E1225Hu	—	0.1–35 µg/mL	0.043 µg/mL	<8%	<10%	2	0
CGA	BT-LAB	E1730Hu	—	30–9000 ng/L	15.21 ng/L	<8%	<10%	2	0
SERPINA1	BT-LAB	E0753Hu	—	0.05–15 mg/mL	0.024 mg/mL	<8%	<10%	1	0
PlGF	BT-LAB	E0138Hu	—	8–1800 ng/L	4.02 ng/L	<8%	<10%	1	0
ApoE	BT-LAB	—	—	10–2000 ng/mL	4.54 ng/mL	<10%	<12%	0	0

BT-LAB, Bioassay Technology Laboratory; ULOQ/LLOQ, upper/lower limit of quantification. For complement factor D the manufacturer advises against sample dilution owing to potential matrix interference. Units: complement factor D, soluble fms-like tyrosine kinase-1, fibronectin and apolipoprotein E in ng/mL; histidine-rich glycoprotein and serum amyloid A in µg/mL; chromogranin-A and placental growth factor in ng/L; α1-antitrypsin (SERPINA1) in mg/mL.

Additional analyses requested by Reviewer 2

Figure S1. Per-outer-fold AUROC for all three classifiers (AdaBoost, XGBoost, CatBoost) across the 5-fold × 3-repeat (15) outer test folds, for each comparison and feature-selection method. The bold value marks the reported best model; the gap to the median classifier quantifies post-hoc model-selection optimism (mean +0.04, up to +0.10).

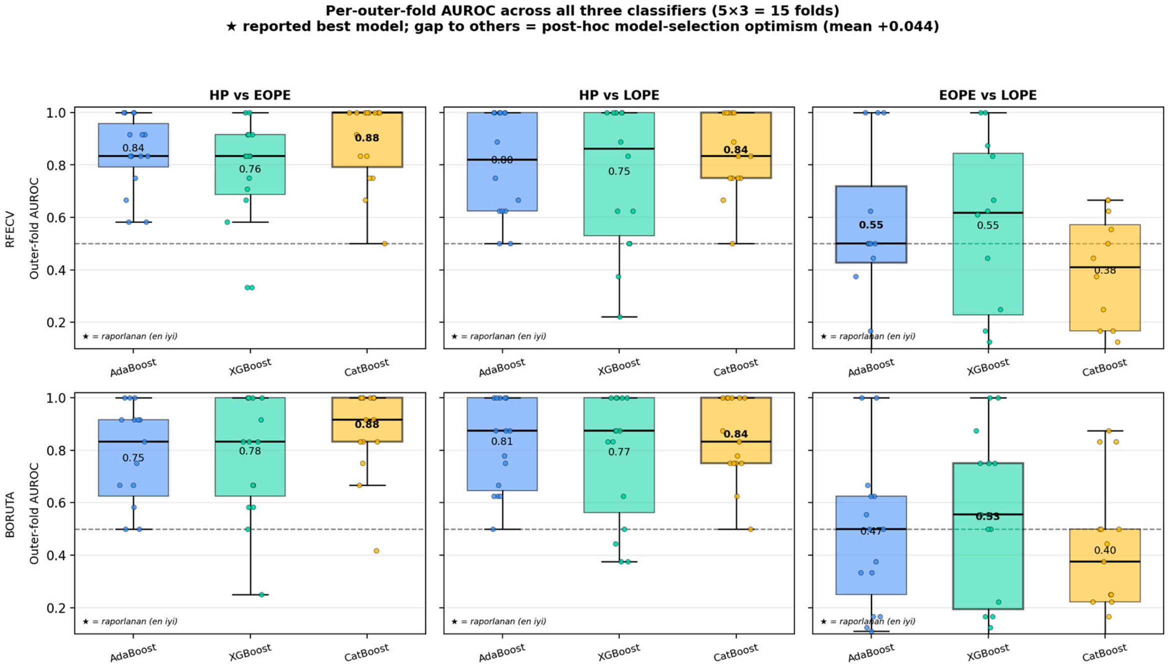

Supplementary Table S6. Benchmarking of each ELISA analyte against soluble fms-like tyrosine kinase-1 (sFlt-1). AUC is the disease-positive area under the curve; DeLong p tests the difference from sFlt-1 on the same participants; ΔCV-AUC is the change in 5-fold cross-validated AUC when the analyte is added to an sFlt-1-only logistic model. Reference sFlt-1 AUC: EOPE–HP 0.82, LOPE–HP 0.67.

**Analyte**	**AUC (EOPE–HP)**	**p vs sFlt-1**	**ΔCV-AUC**	**AUC (LOPE–HP)**	**p vs sFlt-1**	**ΔCV-AUC**
CFD	0.73	0.013	+0.002	0.61	0.099	-0.047
HRG	0.69	0.007	+0.000	0.59	0.120	-0.041
Fibronectin	0.77	0.113	+0.007	0.65	0.520	+0.004
SAA	0.79	0.171	-0.005	0.60	0.031	-0.082
CGA	0.66	0.000	+0.052	0.56	0.008	-0.068
SERPINA1	0.70	0.017	+0.025	0.67	0.952	-0.115
ApoE	0.51	0.001	-0.195	0.39	0.010	+0.039

Supplementary Table S7. Post-hoc power: minimum detectable Cohen d at 80% power (two-sided, α = 0.05) per comparison. TOST equivalence (EOPE vs LOPE): at |log2FC| < 1.0, 38% of proteins equivalent, 3% different, 59% inconclusive (16% / 3% / 81% at |log2FC| < 0.5), so overlap cannot be affirmed over insufficient power.

**Comparison**	**n (per group)**	**d ≥ (α = 0.05)**	**d ≥ (Bonferroni/370)**
EOPE vs LOPE	17 / 11	1.13	2.10
EOPE vs HP	17 / 18	0.98	1.77
LOPE vs HP	11 / 18	1.11	2.06
ELISA validation	29 / 29	0.75	1.31

Supplementary Table S8. Multiple-testing correction (within-comparison vs pooled across all three comparisons, Benjamini–Hochberg; DEP: adjusted p < 0.05 and |log2FC| > 0.5) and selection-stability check. Leading markers and the null early-vs-late result are unchanged under pooling. Selection frequencies exceed a random-selection null (e.g., complement factor D 15/15 folds, Wilson 95% CI 0.80–1.00 vs expected 0.07).

**Comparison**	**within-BH DEP**	**pooled-BH DEP**	**stable > null, BORUTA / RFECV**
EOPE vs HP	10	5	29 / 49
LOPE vs HP	8	3	36 / 38
EOPE vs LOPE	0	0	26 / 64
